# Health Professionals’ Knowledge, Perceptions, and Attitudes Toward Baby-Led Weaning: Scoping Review

**DOI:** 10.1177/23779608241285417

**Published:** 2024-09-27

**Authors:** P. Sarreira–de-Oliveira, S. Fernandes, R. Ramalho, F. Loureiro

**Affiliations:** 1 Egas Moniz School of Health and Science, Egas Moniz Center for Interdisciplinary Research (CiiEM, U4585 FCT), Caparica, Portugal; 2 Egas Moniz School of Health and Science, Nutritional Immunology – Clinical and Experimental Lab (NICE Lab), Clinical Research Unit, Egas Moniz Center for Interdisciplinary Research (CiiEM, U4585 FCT), Caparica, Portugal; 3 Universidade Católica Portuguesa, Faculdade de Ciências da Saúde e Enfermagem, Centro de Investigação Interdisciplinar em Saúde (CIIS), Portugal

**Keywords:** Baby-led weaning, healthcare professional, knowledge, attitudes, perceptions, infant nutrition, review literature, guideline

## Abstract

**Introduction:**

Baby-led weaning (BLW) is a growing method for parents to introduce food to their kids. As advisers who affect the decisions of parents, health practitioners have significant obligations in this regard.

**Objective:**

We aim to identify existing literature on the knowledge, perceptions, and attitudes of health professionals toward BLW.

**Methods:**

We carried out a scoping review in accordance with the Preferred Reporting Items for Systematic Reviews and Meta-Analyses extension for Scoping Reviews. After registering with Open Science Framework, published articles were retrieved from EBSCOhost, PubMed, SciELO, ScienceDirect, Open Grey, and the Portuguese Scientific Open Access Repository. Primary studies with full-text availability in English, Spanish, or Portuguese, and no data publication limitations were included.

**Results:**

The final sample included seven publications conducted in five countries published between 2012 and 2022. Only one study employed a hybrid methodology showing incongruent practices in parents/health professionals on BLW, whereas most studies employed a quantitative approach. Regarding BLW, it is recognized that there is a dearth of evidence, consensus, and advice.

**Conclusion:**

Despite benefits, health professionals hesitate to recommend BLW due to insufficient study and safety concerns, warranting more research. Hence, our scoping review demonstrated that BLW is a scientifically under-researched subject, indicating a significant research gap that must be filled in the future.

## Introduction

The World Health Organization recommends exclusive breastfeeding for the first 6 months of an infant's life, with the addition of complementary feeding for at a minimum of 24 months (WHO, 2021). This 24-month period of a child's life provides an opportunity to ensure proper nutritional intake, prevent malnutrition (Michaelsen et al., 2017), and avoid adverse long-term health consequences (Theurich et al., 2020). There is no unanimity regarding the best pattern of food consumption that should be followed globally (Gatica-Domínguez et al., 2021).

As children transition from a liquid milk-based diet to a solid food-based diet, they are traditionally exposed to a widening diversity of flavors. Usually, complementary feeding begins with spoon-fed puree, then transitions to semisolid and finger meals (Cameron et al., 2012a). Yet, in recent years there has been a trend to skip the early stage and go straight to finger food (Cameron et al., 2012b; Fewtrell et al., 2017). This method is known as baby-led weaning (BLW) and is an alternative to complementary feeding (Arantes et al., 2018). Since no official data has been collected, on people who use this method, the prevalence is unknown. Nonetheless, the amount of scientific literature research has expanded significantly during the previous decade (Brown et al., 2017).

With BLW, children are fed food in modest portions, and they feed themselves during family mealtimes (Fewtrell et al., 2017). It is a technique that provides children with greater control over their food intake, improved appetite control, the development of motor skills (Brown et al., 2017; Wati et al., 2024) and allows children the capacity to control the amount of time it takes them to consume food (Fewtrell et al., 2017). When using this approach, the child's developmental stage must also be considered (Oliveira et al., 2023). Although BLW enthusiasts point to several benefits, health professionals raise several concerns, including the risk of choking, exposure to unhealthier food intake (high-energy and low-nutrient density diets), possible nutrient deficiency or excess, and the overall effect on children's nutritional status and eating behavior (Brown et al., 2017).

## Review of Literature

Previous literature reviews have addressed this topic (Arantes et al., 2018; Bocquet et al., 2022; Brown et al., 2017; Cameron et al., 2012b; D’Auria et al., 2018; Devlin, 2021; Gomez et al., 2020; Martinón-Torres et al., 2021; Scarpatto & Forte, 2018; Utami & Wanda, 2019). Cameron et al. (2012b) evaluated the general literature regarding how feasible BLW might be for parents. They concluded that there was a lack of consensus among scientific community due to the scarcity of studies in this area. Later empirical studies were reviewed to examine behaviors associated to this approach (Brown et al., 2017). Yet, the authors emphasized the need for additional research, even though there is some evidence to suggest that this novel strategy may lead to favorable effects. In 2018 three reviews were performed (Arantes et al., 2018; D’Auria et al., 2018; Scarpatto & Forte, 2018). Arantes et al. (2018) reported that this practice was endorsed by parents who used it; nonetheless, certain problems were mentioned (e.g., mealtime mess and food waste), as well as professionals’ apprehension about children's ability to self-feed. D’Auria et al. (2018) conducted a thorough review of the literature and determined that the available evidence was of poor quality; hence, major issues of BLW must still be addressed. Scarpatto and Forte (2018) assessed literature to compare BLW to the conventional approach and concluded that BLW could be a viable alternative; however, it required medical and/or nutritional monitoring to ensure the infant's nutritional requirements were met. Following, Utami and Wanda (2019) emphasized that BLW was beneficial for fostering infants’ independence, but a paucity of longitudinal trials prevented them from drawing firm conclusions about nutritional sufficiency, intake, eating patterns, food preferences, safety, and growth trends. In the integrative review performed by Gomez et al. (2020) authors emphasized that despite the discovered benefits, there are still significant hazards, such as choking and inadequate vitamin intake, that require additional research. Recently Devlin (2021) undertook a scoping evaluation of 15 studies and concluded that BLW was a safe and effective method for infants’ supplementary feeding. In the same year Martinón-Torres et al. (2021) conducted a systematic review with the objective of establishing the effect of BLW on weight gain compared to traditional methods. The authors observed inconsistent outcomes, with some research suggesting a decreased weight with the use of BLW and others showing equivocal results. Again, the authors underlined the need for additional research. The last review found (Bocquet et al., 2022) was performed by the Nutrition Committee of the French Pediatric Society. Although benefits of the BLW strategy were identified (breastfeeding promotion, respect for children's appetite, and use of unprocessed meals, among others), the disadvantages (risk of not getting enough energy, iron, zinc, vitamins, and other nutrients, or getting too much protein, saturated fat, salt, or sugar, and risk of choking) prevented them from recommending it over the conventional one.

In the literature, studies have been identified that address parents’ perspectives on BLW or BLW steps (Arslan et al., 2023; Białek-Dratwa et al., 2022; Boelsma et al., 2021). Despite the importance of this topic, less research appears to have been conducted on the knowledge, perceptions, and attitudes of health professionals (Arantes et al., 2018). However, this issue is important because health professionals provide advice and support to parents and are a valuable source of information that can influence parents’ decisions about their children (Boelsma et al., 2021). Few of the identified reviews consider the perspective of health professionals, although they are an important part of this topic. Therefore, we conducted a scoping study that aims to synthesize the current knowledge, perspectives, and attitudes of health professionals regarding BLW. Due to its usefulness for surveying a body of research, a scoping review was favored above other types of literature reviews (Munn et al., 2018).

## Methods

Initially a preliminary search was conducted on different platforms namely: PubMed; PROSPERO; the Cochrane Database of Systematic Reviews; the Joanna Biggs Institute Evidence Synthesis; FigShare; and the Open Science Framework. No ongoing scoping review or scoping protocol that assessed knowledge, perceptions, and attitudes of health professionals toward BLW was located. This is a gap in the literature and there is a need to map knowledge in this specific area, given the influence of health professionals on parents’ choices and the growing interest in this topic.

The research question was defined according to PCC (Population, Concept, Context): what is the knowledge, perceptions, and attitudes of health professionals toward BLW? (Population: health professionals; Concept: BLW; Context: children care). Scoping review steps followed Tricco et al. (2018) recommendations as detailed bellow.

### Protocol and Registration

This scoping review protocol was drafted according to Preferred Reporting Items for Systematic Reviews and Meta-Analyses extension for Scoping Reviews (PRISMA-ScR) (Tricco et al., 2018) and registered in Open Science Framework in January 2023 (https://osf.io/g3a2u/).

### Eligibility Criteria

Consideration was given to manuscripts addressing the knowledge, perspectives, and/or attitudes of health professionals toward BLW. Empirical studies were included regardless of the methodology employed (qualitative, quantitative, or mixed methods). The search was restricted to full-text, freely accessible articles published in English, Spanish, or Portuguese. To maximize the breadth of the available evidence, no data restrictions were imposed. Articles with a sample consisting of both parents and health professionals were included if the analysis allowed the health professional perspective to be clearly distinguished. Methodological research, proceedings, literature reviews, editorials, blog posts, advertising, and opinion pieces were excluded.

### Information Sources

To identify the relevant keywords for the search equation, a preliminary search was conducted on Medical Literature Analysis and Retrieval System Online (MEDLINE) and Cumulative Index to Nursing and Allied Health Literature (CINAHL). Secondly the research was performed in CINAHL complete; MEDLINE complete; Nursing & Allied Health Collection (comprehensive); Cochrane Central Register of Controlled Trials; Cochrane Database of Systematic Reviews; Cochrane Methodology Register; Cochrane Clinical Answers; Library, Information Science & Technology Abstracts (LISTA); eBoook University Press collection; eBook Collection; Teacher Reference Center; Education Resource Information Center (ERIC); and MedicLatina via EBSCOhost platform. Additionally, search was also conducted in PubMed, SciELO, SCOPUS, Embase, OpenAIRE, and ScienceDirect. Open Grey, Mednar, B-on, and Portuguese Scientific Open Access Repository (RCAAP) were used for grey literature search. As a third step the list of references from previous literature reviews and the selected article were searched to detect additional relevant literature.

### Search

Different keywords (baby led weaning; BLW; health professionals; knowledge; attitudes; perceptions) were combined with Boolean operators (AND / OR) in the research equation without camp limitation. The search formula was the following: ((knowledge OR perception OR attitude) AND (health professionals OR health personnel OR health care provider) AND (baby led weaning OR BLW OR baby-led)). Since platforms have distinctive characteristics different grouping and combinations were used. The search strategy is available on the Open Science Framework and was performed by all authors working in two groups of two in November 2023, with an update in January 2024. After group working, a more consensual outcome was achieved by networking of all authors.

### Selection of Sources of Evidence

Duplicates were removed from our initial article sample, and inclusion/exclusion criteria were applied. The selection was based on the article's title, and the abstract was read if it was unclear whether the study was relevant to the review. All authors reviewed the same papers to ensure uniformity. In cases of disagreement between researchers, the articles acceptability was handled through debate until consensus was reached.

### Data Sharting Process

A data graphing table was created in Microsoft Excel® to extract variables. All authors collaborated on a shared document and discussed the items to add in the data graphing table to complete the procedure. Then, each researcher extracted the data independently and afterwards compared it. To improve precision, disagreements were handled through discussion until a consensus was formed.

### Data Items

Characteristics from each article were extracted concerning the following items: authors, year, country, purpose, methods, and main findings. These items were chosen by all authors as the most relevant ones to identify main studies characteristics.

### Critical Appraisal of Individual Sources of Evidence

Article quality was appraised individually by each author and then discussed until agreement. For critical appraisal the Hawker et al. (2002) assessment tool was used. The tool has a four-grade scale (1 = very poor; 2 = poor; 3 = fair; 4 = good) and evaluates articles quality based on items such as: abstract and title; introduction and aims; method and data among others. The total tool score ranges from 9 to 36, and higher scores indicate a higher article quality.

### Synthesis of Results

Synthesis of results was concentrated in a table that combined all extracted information. This table was discussed and approved by all authors. Collected data summarized studies characteristics and gave an overview of main findings.

## Results

### Selection of Sources of Evidence

**Figure 1.**
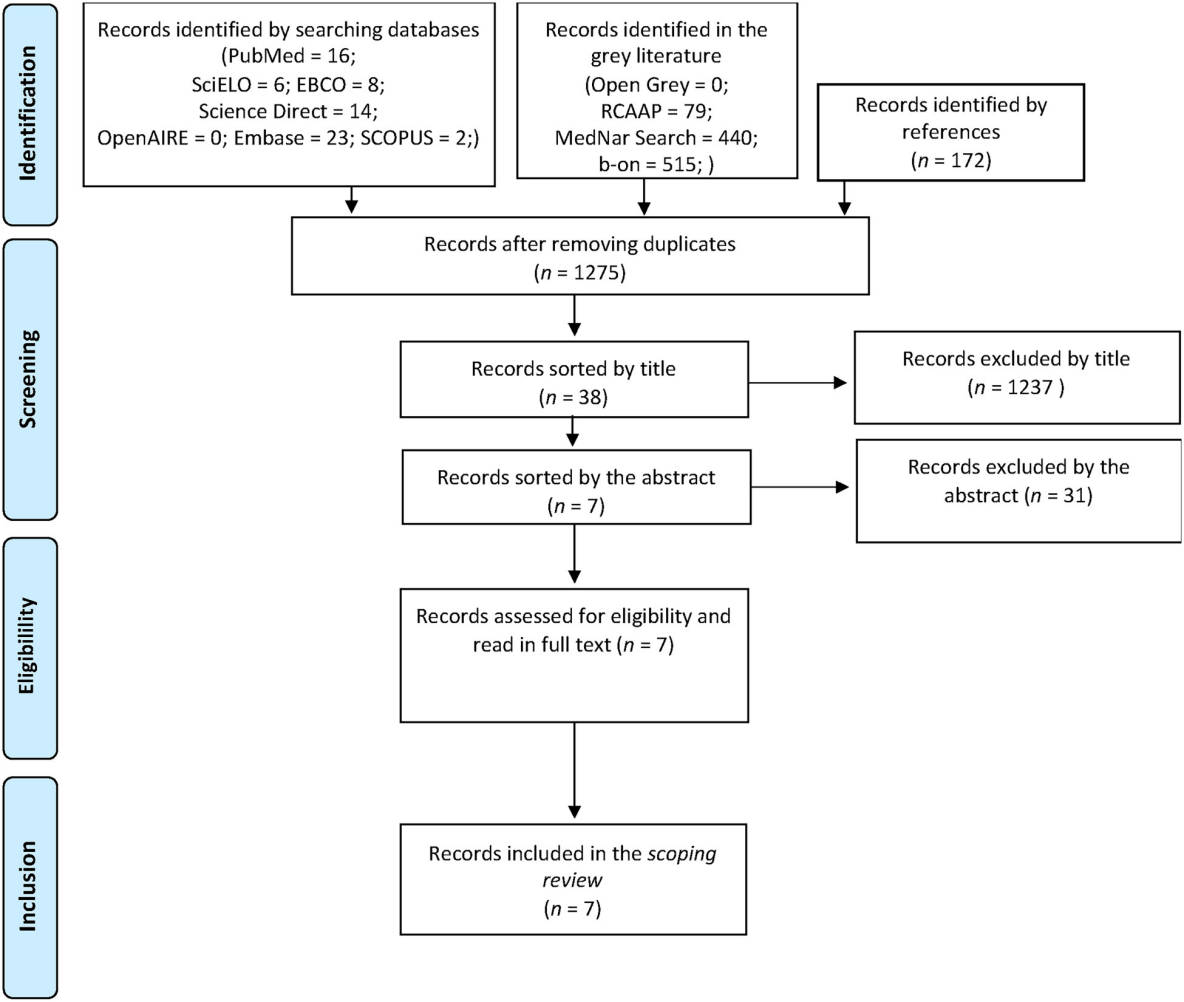
PRISMA flow chart of study selection.

From our initial sample of 1275 a total of seven articles were selected and included in this review. Articles were excluded based on title because they were outside the scope of this review (1200) or the sample was only parents/caregivers (37). Afterwards abstract was scanned and 31 articles were excluded because health professionals were not included in the sample. Study selection process is summarized in a flow chart adapted from PRISMA in Figure 1.

### Characteristics of Sources of Evidence

Publication dates from our article sample ranged from 2012 to 2022. Studies were performed in France (de Rosso et al., 2022), Spain (Arias-Ramos et al., 2022; Rubio et al., 2018; Mauro Martín et al., 2022), New Zeeland (Cameron et al., 2012a), Canada (D’Andrea et al., 2016), and Brazil (Neves et al., 2022). Most studies used a quantitative approach (D’Andrea et al., 2016; Neves et al., 2022; Rubio et al., 2018; Mauro Martín et al., 2022) and only one study (Arias-Ramos et al., 2022) applied a mixed method approach. Sample size ranged from 866 (Mauro Martín et al., 2022) to 21 (de Rosso et al., 2022) and questionnaires (Arias-Ramos et al., 2022; D’Andrea et al., 2016; Neves et al., 2022; Rubio et al., 2018; Mauro Martín et al., 2022) were the most common method of data collection used. Table 1 synthetizes the main studies characteristics.

**Table 1.** Study Characteristics.

Author, year, and country	Objective	Methods	
		Type of study and data collection method	Sample
Arias-Ramos et al., 2022, Spain	To describe the knowledge of health professionals about complementary feeding and BLW method and the attitudes toward its recommendation and to explore the knowledge, experiences and attitudes of parents who have used this method	Mixed methods, questionnaires, and semistructured interviews	n = 48; HCP (doctors, nurses, midwives; n = 38) and mothers (n = 10)
Cameron et al., 2012a, New Zealand	To examine healthcare professionals’ and mothers’ knowledge, attitudes and experiences regarding BLW	Qualitative, semistructured interviews	n = 51; HCP (practice nurses, well-child providers, dietitians, general practitioners, pediatricians, lactation consultants, midwives, and pediatric speech-language therapist; n = 31) and mothers (n = 20)
D'Andrea et al., 2016, Canada	To compare common BLW practices and associated knowledge and perceptions of practicing mothers and HCP	Quantitative, questionnaires	n = 98; HCP (dietitians, nurses, lactation consultants, physicians, occupational therapist, physiotherapist; n = 33) and mothers (n = 65)
de Rosso et al., 2022, France	To assess professionals’ practices and perceptions regarding their communication with parents on child feeding and to evaluate their perception of the draft of the new brochure	Qualitative, online semistructured interviews	n = 21; Pediatricians and general doctors’ practitioners (n = 13); healthcare/childcare professionals (n = 8)
Martínez Rubio et al., 2018, Spain	To assess the attitudes of Spanish primary care pediatricians regarding complementary feeding and review the published evidence on the BLW approach	Quantitative, questionnaires	n = 579; Primary care pediatricians
Mauro Martín et al., 2022, Spain	To evaluate the knowledge and attitudes of a group of parents and HCP regarding BLW in Spain	Quantitative, questionnaires	n = 866; HCP (nurses, doctors, nutritionists, and pharmacists; n = 364) and parents (n = 502)
Neves et al., 2022, Brazil	To describe Brazilian health professionals’ perception about the BLW method use for complementary feeding	Quantitative, questionnaires	n = 458; HCP (nursing, speech therapy, medicine, nutrition, and dentistry)

*Note*. BLW=baby-led weaning; HCP=healthcare professionals.

### Critical Appraisal Within Sources of Evidence

As stated earlier critical appraisal was performed according to Hawker et al.’s (2002) recommendations. Individually, each author performed the critical evaluation, and disagreements were resolved through discussion and consensus. Overall, quality appraisal was quite high and ranged from 28 to 31 as shown in Table 2.

**Table 2.** Studies Quality Appraisal.

Study	Abstract and title	Introduction and aims	Method and data	Sampling	Data analysis		Ethics and bias	Results	Transferability or generalizability	Implications and usefulness	Total
Rubio et al., 2018	3	3	4	3	3		2	4	3	3	28
de Rosso et al., 2022	4	3	4	3	4		3	4	3	3	31
Cameron, Heath & Taylor, 2012a	4	3	4	3	4		3	4	3	3	31
D’Andrea et al., 2016	4	3	4	3	4		3	4	3	3	31
Mauro Martín et al., 2022	4	3	4	3	4		3	4	3	3	31
Neves et al., 2022	4	3	4	3	4		3	4	3	3	31
Arias-Ramos et al., 2022	4	3	4	3	4		3	4	3	3	31

### Results of Individual Sources of Evidence

Since our aim was to synthetize current information, we retrieved the relevant data on knowledge, perceptions, and attitudes of health professionals toward BLW, namely the main findings from each study as synthetized in Table 3. Most studies combined different concepts such as knowledge, attitudes, and experiences (Arias-Ramos et al., 2022; Cameron et al., 2012) or knowledge, practices, and perceptions (D’Andrea et al., 2016). Only two articles aimed only one concept namely attitudes (Rubio et al., 2018) and perceptions (Neves et al., 2022).

**Table 3.** Studies Main Findings.

Study	Main findings	Instrument
de Rosso et al., 2022	Professionals mentioned that they face an increasing number of questions about BLW and need more evidence-based information	Not applicable
D'Andrea et al., 2016	HCPs stated that knowledge about BLW came from another HCP, a client, or through professional development. When compared mothers believed more that BLW encouraged healthier eating and prevented fussy/picky eaters than HCPs. Most mothers and HCPs believed that BLW promoted fine and oral motor skill development and shared family meals. Increased risk of infant choking, low energy and iron intake, and increased maternal competition were identified by HCP as disadvantages. The impact of social media, online forums, like the Facebook group, provides an excellent opportunity to support and educate mothers and therefore HCPs should consider them	Questionnaire designed for this study (questions adapted from other study [Cameron et al., 2012b])
Mauro Martín et al., 2022	Most HCP knew about the BLW model, its benefits and had accompanied families who followed BLW. Most professionals agree that BLW facilitates the transition to family feeding, makes it easier for the baby to adapt to new flavors and consistencies, enhances chewing against sucking and can promote the maturational development of the baby. Likewise, all health professionals, except for nonpediatric doctors, consider that BLW favors the development of fine motor skills. In contrast, all professionals disagreed with babies not gaining enough weight or getting deficiency in some nutrients	Adapted questionnaire (Rubio et al., 2018)
Cameron, Heath & Taylor, 2012a	Nearly half HCP knew about BLW and had been introduced to the concept by their colleagues, friends, and family. HCP considered that BLW could be beneficial and improved shared family mealtimes. Strong concerns about BLW were also expressed: choking, dietary disadvantages, and food wasted	Not applicable
Neves et al., 2022	Most professionals reported knowing BLW, however only a 1/5 usually recommend this approach. BLW was reported as advantageous, but disagreements were evidenced (comfort/convenience and the argument of generating less concern or anxiety for parents/caregivers)	Adapted questionnaire (Arantes et al., 2018; Cameron et al., 2012b; D’Andrea et al., 2016; Rubio et al., 2018)
Arias-Ramos et al., 2022	Most of the HCP are aware of the BLW method and its benefits however, they do not recommend it in all cases (concerns about choking, low-energy intake, little scientific evidence or the risk of an unhealthy diet). Mothers consulted mainly informal sources of information to learn about and implement it. The mothers noted that the method was natural, encouraged infant autonomy, and promoted healthy eating habits. They listen to the advice of professionals but continue to rely on other informal sources of information	Adapted questionnaire (Rubio et al., 2018)
Martínez Rubio et al., 2018	79% of HCP knew about BLW, 45.3% recommended it in some cases and 6.6% routinely. The main concerns of the respondents were lack of information (67.2%), lack of scientific evidence (10.6%), and the potential risk of choking (10.6%)	Questionnaire designed for this study

*Note*. BLW=baby-led weaning; HCP=healthcare professionals.

### Synthesis of Results

Only seven articles were included in the final sample. All articles assessed health professionals or both parents and health professionals’ knowledge, perceptions, and attitudes on BLW. Although benefits are recognized the lack of reliable studies seems to be a major concern for professionals. Main results were synthetized in tables to facilitate reading and information organization. In addition, we used the PAGER framework (Bradbury-Jones et al., 2021) to summarize our key findings, as detailed in Table 4.

**Table 4.** Synthesis of Review Findings Under the PAGER Framework.

Patterns	Advances	GAPS	Evidence for practice	Research recommendations
The BLW method is increasingly used by parents who seek advice from health professionals	The number of healthcare professionals familiar with this method and its benefits is growing	There are no clear statistics on the use of BLW by parents or on the recommendations of the method by healthcare professionals	Systematic registration of the use and implementation of this method should be promoted by health organizations	Development of national studies for the assessment of the current use of the BLW method
Healthcare professionals report insufficient understanding about BLW	Healthcare providers identify a lack of knowledge and doubt about the steps to use this method, its benefits, and major constraints	It is not clear to what extent health professionals know about this method	Data collection on BLW knowledge and practices of health professionals should be undertaken by health organizations as a first step in planning education on this topic	Development and validation of instruments to assess the knowledge of health professionals on this topic
Healthcare professionals report a lack of clear guidelines for use of BLW	Healthcare professionals identify three main dimensions: benefits, constraints, and steps	Each of these areas (benefits, constraints, and steps) is under-researched	Education and training in the BLW method should be on the agenda of healthcare professionals and healthcare organizations	Development of structured programs on the BLW method for health professionals working in the field of child nutrition Integrate this method into the undergraduate nursing course to ensure basic knowledge of BLW for new nursing graduates Committees and organizations need to establish clear guidelines on the steps for using BLW

*Note*. BLW=baby-led weaning.

## Discussion

### Summary of Evidence

Seven articles met inclusion criteria after a thorough platform and database search, demonstrating a lack of evidence on this topic. Although the number of studies we were able to find is small, they are spread across several countries (Spain, New Zealand, Canada, France, and Brazil) and were published between 2012 and 2022. Even scarce, these studies indicate a tendency for interest in this topic to spread geographically in scientific communities. We found that research’ methods for assessing health professionals’ knowledge differed. Most studies assess health professionals’ knowledge (Arias-Ramos et al., 2022; Cameron et al., 2012b; D’Andrea et al., 2016; Mauro Martín et al., 2022), some combined attitudes assessment (Arias-Ramos et al., 2022; Mauro Martín et al., 2022) or only attitudes (Martínez Rubio et al., 2018). Only two evaluated perceptions (de Rosso et al., 2022; Neves et al., 2022) and two measured practices and knowledge (D’Andrea et al., 2016) or perceptions (de Rosso et al., 2022). Most importantly, the concept under evaluation was rarely addressed. This may influence the observation and measurement of variables, which we considered an important bias. Future studies should begin by clearly stating the concept evaluated early (Polit & Beck, 2017).

Nurses, midwives, well-child providers, dietitians/nutritionists, general practitioners, pediatricians, lactation consultants, pediatric speech-language therapist, occupational therapist, physiotherapist, pharmacists, and dentists were assessed. The professional groups’ contributions are diverse and rich. In contrast, such heterogeneity makes it difficult to establish precise and uniform information. Having a broad group advice parents on their children's nutrition can be challenging.

Three key themes emerge from this review: the BLW approach has become increasingly popular with parents who seek advice from health professionals; health professionals acknowledge a lack of comprehensive understanding of BLW; and there is a need for guidelines on the use of BLW. All publications state that the use of this method has not been properly studied for professional associations or international committees to approve it and provide guidelines to guide the practice of health professionals. However, the method is used by parents who seek advice from professionals (Arias-Ramos et al., 2022), but also from multiple sources (e.g., support groups, informal groups, and online resources), sometimes with conflicting information (Prescott, 2024). Therefore, education and increased research on BLW appears as a major necessity for health professionals.

Main advantages identified by health professionals include easier transition to family shared feeding (Cameron et al., 2012b; Neves et al., 2022; Mauro Martín et al., 2022), better adaptation to new flavors and consistencies (Mauro Martín et al., 2022), enhanced children development (Arias-Ramos et al., 2022; Cameron et al., 2012b; D’Andrea et al., 2016; Mauro Martín et al., 2022), and healthier food behaviors (Arias-Ramos et al., 2022). However, concerns are also identified with an important weight on professionals’ recommendation on BLW. All studies report lack of evidence on this approach. Other concerns include risk of choking (Arias-Ramos et al., 2022; Cameron et al., 2012b; D’Andrea et al., 2016; Martínez Rubio et al., 2018), insufficient nutrient intake (Arias-Ramos et al., 2022; Cameron et al., 2012b; D’Andrea et al., 2016; Mauro Martín et al., 2022), and as food wasting (Cameron et al., 2012b). However, professionals report an increase in BLW questions and a lack of reliable information. These findings support the limited percentage of professionals who routinely recommend BLW (Martínez Rubio et al., 2018; Neves et al., 2022).

Parents search the internet, especially social media, for information. This information may be unreliable and unscientific (Voloshyn et al., 2023). Healthcare professionals should consider using this resource to communicate knowledge, given its importance in this field (D’Andrea et al., 2016). Parents are exploring this issue despite limited data, and this review identified a gap in knowledge about the perspectives of healthcare professionals.

## Strengths and Limitations

Strengths of this review include a comprehensive synthesis of the literature published on this topic, as well as a summary of implications for practice and future research. By identifying articles that assessed health professionals’ knowledge and practice of the BLW method, we were able to contribute with a synthesis of what is already known and what should be addressed by future research.

We must regard the lack of studies in this research area to be a significant limitation. Although we performed a wide search for relevant studies only seven were included. This fact impacted our search and underscored the need to invest in this area. In addition, we only included articles with open access, which is also a limitation. Furthermore, the studies found included samples of different sizes, which does not allow consistent conclusions to be drawn, which is also a limitation.

## Implications for Practice

Based on our findings, research recommendations include conducting national studies to evaluate the current use of the BLW method, creating and validating instruments to assess health professionals’ knowledge of this topic, creating structured programs for health professionals working in the field of child nutrition to learn about the BLW method, incorporating this method into undergraduate nursing courses to ensure that new nursing graduates have a basic understanding of the BLW, and establishing clear guidelines for the use of the BLW by committees and health organizations.

## Conclusions

We identified seven 2012–2022 articles on health professionals’ attitudes, perceptions, knowledge, and practices in five countries for this scoping review. After analyzing the articles, we determined that the proportion of specialists that frequently advocate the BLW method is quite low. Health professionals acknowledge that BLW has benefits, such as an easier transition to shared family meals, better adaptation to new tastes and consistencies, improved child development, and healthier eating behaviors, however the implementation of this method has not yet been sufficiently studied for professional committees to recommend it. The articles point to a lack of scientific evidence for this method and raise questions about its recommendation, including the risk of choking, inadequate nutrient intake, and food waste. We also found that BLW is a method increasingly used by parents who seek advice from health professionals. It is therefore essential to increase research and education in this area. Only then can these professionals feel grounded enough to provide the necessary support and guidance that parents are seeking. This scoping review has clearly demonstrated that BLW is a scientifically underexplored topic, indicating a significant research need to be addressed in the future.

## Supplemental Material

sj-docx-1-son-10.1177_23779608241285417 - Supplemental material for Health Professionals’ Knowledge, Perceptions, and Attitudes Toward Baby-Led Weaning: Scoping Review Supplemental material, sj-docx-1-son-10.1177_23779608241285417 for Health Professionals’ Knowledge, Perceptions, and Attitudes Toward Baby-Led Weaning: Scoping Review by P. Sarreira–de-Oliveira, S. Fernandes, R. Ramalho and F. Loureiro in SAGE Open Nursing
